# Cholesterol Granuloma From a Developmental Odontogenic Cyst: A Report of a Rare Case and a Literature Review

**DOI:** 10.7759/cureus.54545

**Published:** 2024-02-20

**Authors:** Ashwin Pattabhi, Sneha Pendem, Dharini S, Monal Yuwanati, Murugesan Krishnan

**Affiliations:** 1 Oral and Maxillofacial Surgery, Saveetha Dental College and Hospitals, Saveetha Institute of Medical and Technical Sciences, Saveetha University, Chennai, IND; 2 Oral Pathology and Microbiology, Saveetha Dental College and Hospitals, Saveetha Institute of Medical and Technical Sciences, Saveetha University, Chennai, IND

**Keywords:** middle ear cholesteatoma, odontogenic tumor/cyst, dentigerous cyst (dc), cholesterol crystals, cholesterol granuloma

## Abstract

Dentigerous cysts are the second most common developmental odontogenic cysts that develop around the crown of unerrupted teeth with the maxillary canine region being one of the common sites of occurrence. The cystic lining of this lesion has been shown to develop into ameloblastoma, Muco epidermoid carcinoma, and squamous cell carcinomas. However, the development of cholesterol granuloma (CG) in the cystic lining of a dentigerous cyst is extremely rare. CG is a histological observation distinguished by the presence of a conglomeration of connective tissue and granulation tissue. The condition is predominantly seen in the field of otolaryngology, with very few cases reported in the maxillofacial region, most of which are associated with the maxillary sinus. This article presents the findings of a CG in a 39-year-old male patient that developed within the dentigerous cyst and discusses the possible etiopathogenesis, surgical management, and histological presentation.

## Introduction

Dentigerous cysts are the most common developmental odontogenic cysts, comprising approximately 20% of all epithelium-lined cysts in the jaws, as evidenced around the crown of an impacted or unerupted tooth. Mandibular and maxillary third molars, followed by the maxillary canine teeth, are common sites for the occurrence of the aforesaid condition [1]. Clinically, they are slow-growing pathological entities; however, the presence of metaplastic changes in the cystic lining often leads to rapid growth. Unicystic ameloblastoma, mucoepidermoid carcinoma, and intra-intraosseous squamous cell carcinoma appear to be the common neoplastic lesions evidenced to originate from the cystic lining of the dentigerous cyst. Histologically, the cyst is lined by a few layers of epithelium that correspond to the reduced enamel epithelium. However, rarely, inflammation of the lining of the cystic wall can lead to the deposition of cholesterol crystals, hemosiderin, and Rushton's hyaline structures within the cyst wall, which are identical to the ones seen in radicular cysts [2]. One of the rarer sequelae of these changes is the development of cholesterol granulomas (CGs).

CGs are a rare, benign pathological entity composed of fluids, lipids, and cholesterol crystals and are surrounded by fibrous connective tissues. These often involve the maxillary antrum and the frontal sinuses. A few cases of CGs have been reported to occur in the walls of inflammatory odontogenic cysts. However, their occurrence in developmental odontogenic cysts has rarely been reported, with CGs often being associated with odontogenic inflammatory cysts [3].

The term "cholesterol granulomas" is often applied interchangeably with cholesteotomas. Cholesteatomas are often seen in the middle ear, secondary to otitis media that is caused by the ingrowth of the squamous epithelium into the middle ear like an epidermoid cyst. Understanding this difference in pathogenesis is essential to distinguish this lesion from CGs, as cholesteatomas are devoid of cholesterol deposition or crystallization, which is the primary pathological finding in CGs. Thus, the current paper aims to describe a case of CG from the cystic lining of a dentigerous cyst and discuss in detail its etiopathogenesis, management, and prognosis [4].

## Case presentation

**Figure 1:**
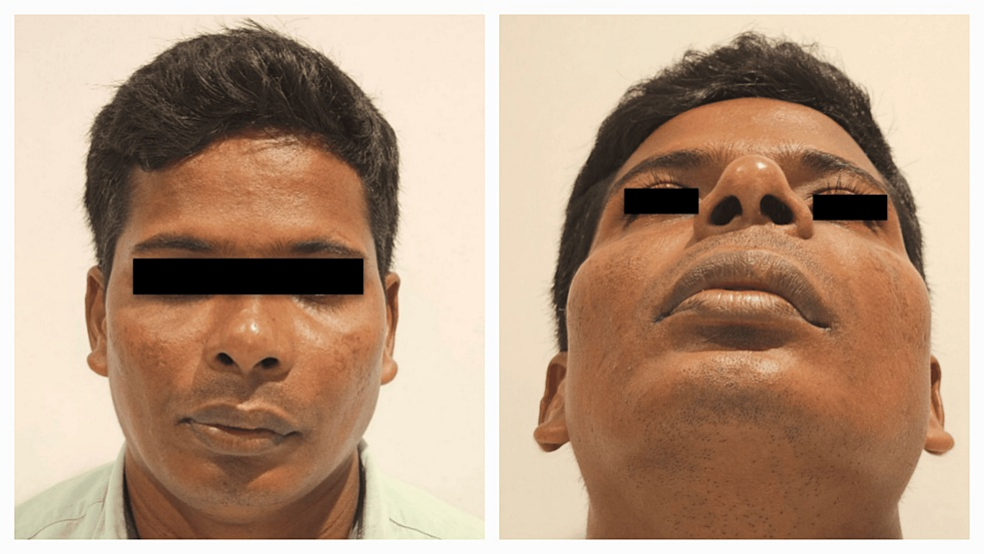
Preoperative frontal and worm's eye views

A 39-year-old male patient with no known comorbidities presented to the Department of Oral and Maxillofacial Surgery with a complaint of swelling and pain in the left maxilla for six months' duration with associated difficulty in deglutition and speech (Figure 1).

A clinical examination of the patient revealed a well-circumscribed swelling measuring 5 x 3 cm that was tender, non-compressible, non-reducible, fluctuant, and soft in consistency. There was healthy mucosa over the swelling, and there were no signs of pus discharge or bleeding. A dentition examination revealed that tooth 25 was clinically missing.

**Figure 2:**
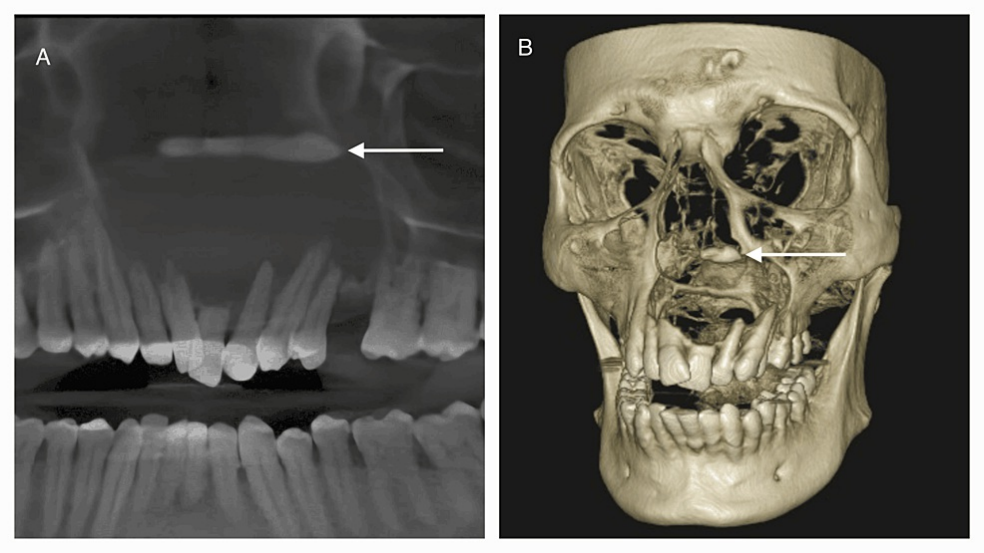
Radiographic images: A) Curved slicing of the cone beam computed tomography showing a unilocular well-defined radiolucency in the periapical region of 13 to 26 involving the nasal floor. B) 3D reconstructed CBCT image showing the radiographic defect and presence of the impacted tooth in the nasal floor (white arrow).

A cone beam computed tomography (CBCT) was carried out, which revealed a well-defined, unilocular radiolucency with a thin sclerotic margin measuring 5 x 3 cm in size, closely associated with an impacted tooth in the nasal floor. Following the CBCT scan, an aspiration of the swelling was initially done, and a chocolate-colored aspirate was obtained and given for cytology. The histopathology report for the cytology revealed mixed inflammatory cells showing plasma and foamy macrophages, revealing an inflammatory cyst. A subsequent incisional biopsy of 0.6 x 0.4 mm was taken from the anterior maxilla, which reported evidence of an odontogenic epithelial lining of two- to four-cell thickness (Figure 2).

**Figure 3:**
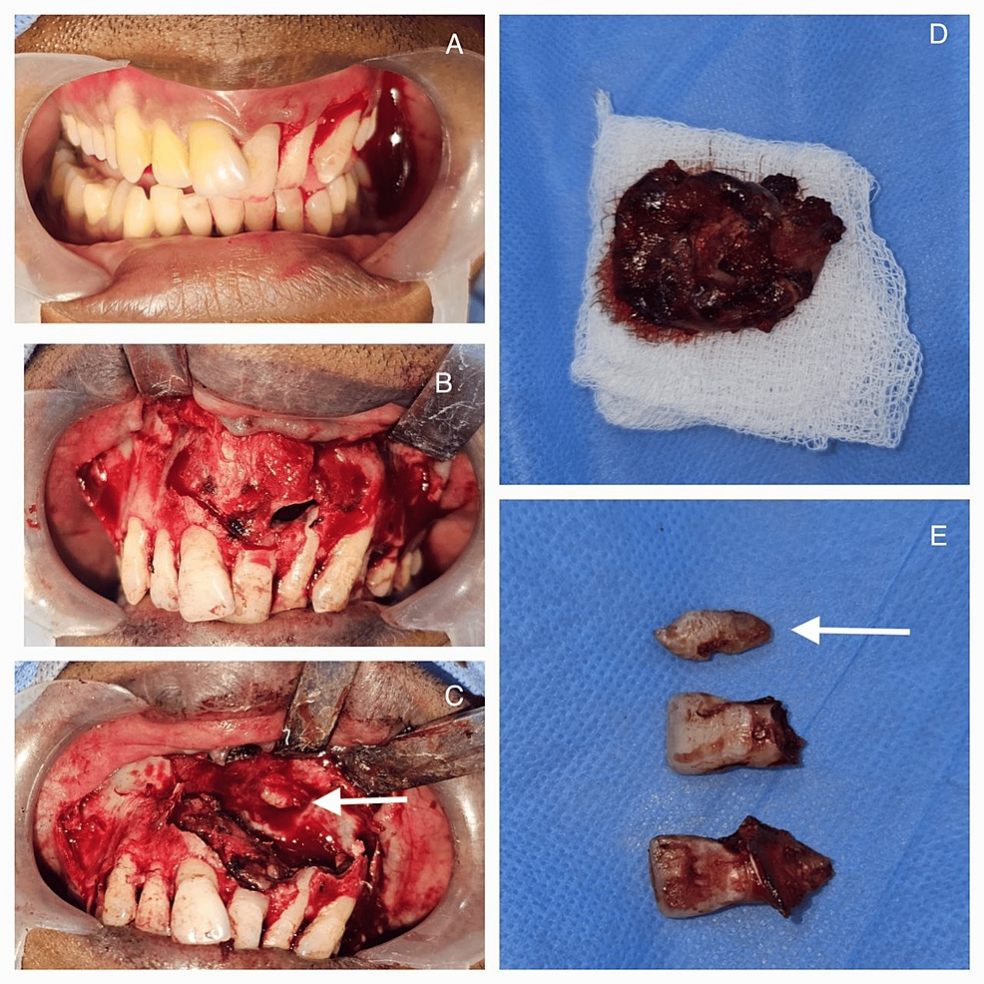
Intraoperative images: A) Intraoral image of the patient showing the buccal aspect of the lesion. B) Full-thickness mucoperiosteum flap raised showing a necrotic eroded bone in the buccal aspect of the maxilla. C) Presence of the impacted teeth in the nasal mucosal floor. D) Excised specimen. E) Impacted teeth along with the extracted 11 and 21 (white arrow).

**Figure 4:**
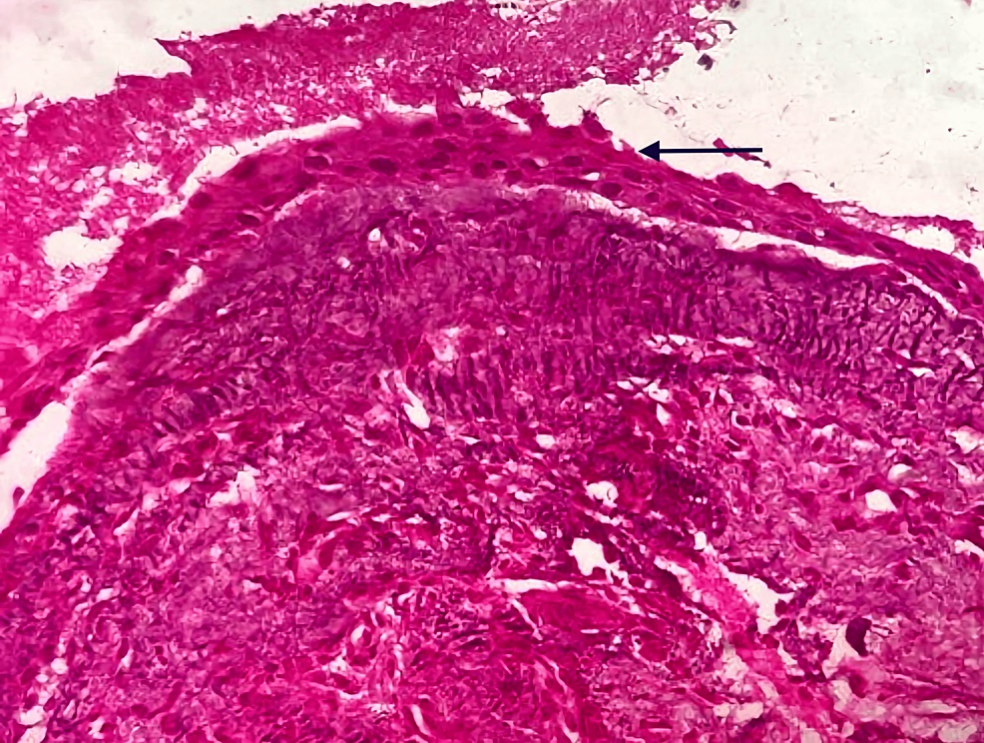
Histopathological examination of the incisional biopsy: odontogenic epithelial lining with two- to four-cell thickness (black arrow)

**Figure 5:**
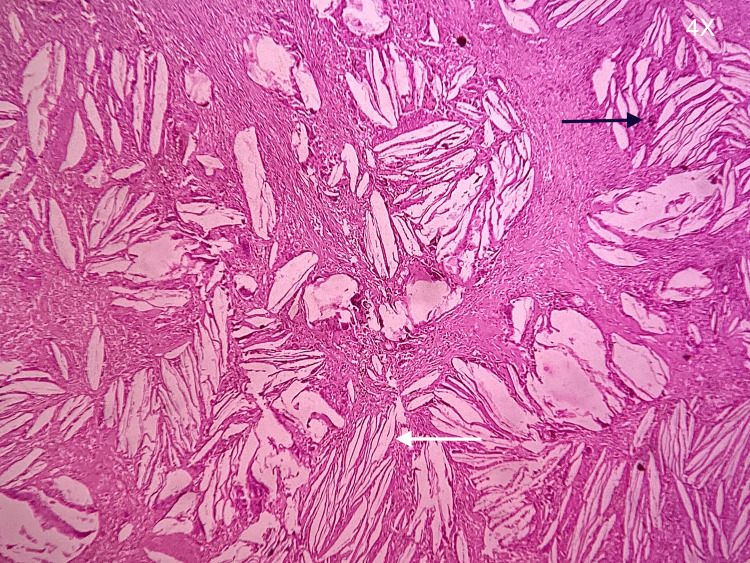
Histopathological examination of the excisional biopsy: A substantial assemblage of longitudinal fissures, characterized by the presence of cholesterol crystals (white arrow) enveloped by foreign body giant cells and macrophages containing hemosiderin (black arrow).

The patient was taken for surgical enucleation and curettage of the cyst under general anesthesia. A buccal crevicular incision was placed from 13 to 26 with a releasing incision, and a full-thickness mucoperiosteal flap was raised. The bone overlying the cyst was removed, exposing the cystic lining. Careful cyst enucleation was done in the superior aspect to identify the impacted tooth close to the nasal floor. The tooth was removed, and the cyst was enucleated in toto (Figure 3). The excised lesion was subjected to histopathological examination. The histopathological report of the tissue showed circumscribed dense fibrous connective tissue with numerous cholesterol clefts and associated multicellular giant cells (Figure 4). There was also a non-keratinized stratified squamous epithelial lining of predominantly two- to four-cell layer thickness (Figure 5). The periodic acid-Schiff (PAS) special staining was negative. The final histopathological diagnosis was suggestive of a CG associated with a dentigerous cyst around the impacted 25 after correlation clinically and radiographically.

Postoperatively, the patient was discharged on the second postoperative day and was given routine postoperative instruction, and a follow-up review was done on the seventh post-operative day, which showed satisfactory wound healing with no clinical complication.

## Discussion

A CG is a rare pathological entity that is often confused with cholesteatoma. The former is a granulomatous lesion associated with the cystic lining common in odontogenic cysts, caused due to the accumulation of cholesterol deposits from the cell membrane. This in turn stimulates a macrophage response that endocytoses the fat and forms foamy cells, and their lysis leads to the release of cholesterol into the extracellular compartment, leading to its crystallization and precipitation. This cholesterol dissolves in the fixative solution, leading to the formation of cholesterol clefts. This is in contrast to cholesteatoma, which is seen in the middle ear and rarely in the paranasal sinuses and is formed due to the invasion of the middle ear by the squamous epithelium from the external auditory canal. These lesions can be histologically differentiated by the presence of an epithelial lining in the latter. It is important to distinguish the latter from the former, as the latter has the potential for recurrence with a potential for malignant transformation [1,2].

CGs are rare pathological entities that are seldom seen in the walls of inflammatory cysts. Incidence in the lining of the developmental odontogenic cyst is extremely rare, and only 13 cases have been reported so far. They could potentially develop from an odontogenic cyst as a response to cholesterol crystals. Inflammatory cysts, particularly radicular cysts, are the primary location for the presence of cholesterol crystals. On the other hand, CGs have been found to have a comparatively low occurrence of developing cysts, such as odontogenic keratocysts. Indeed, every documented instance of CGs originating in the maxilla was associated with the maxillary sinus, with the exception of a single case involving an odontome located in the palate [3].

Gunes et al. presented the first research that examined CGs at the molecular level [4]. They proposed a potential link between perlecan, a substance found in the cystic wall of immature granulation tissue, and the formation of CGs [4]. The detailed pathogenic mechanism for the development of CGs starts with low-density lipoprotein entrapped by perlecan followed by accumulation and oxidation of perlecan in extracellular spaces, which are scavenged by macrophages, and conversion of macrophages into lipid-laden foamy cells takes place, followed by the rupture of these foamy cells, which lead to the release of lipid concentrates into the extracellular space and thereafter crystallization of the concentrated free cholesterol. Foreign body reaction of the cholesterol crystals takes place, which extends inflammatory reaction and promotes cystic growth.

The majority of reports regarding CGs are associated with cysts associated with the maxillary sinus, which makes this case peculiar due to the etiological factor being an impacted tooth and having a developmental origin [5,6].

The histopathological reports of the lesion showed well-circumscribed dense fibrous connective tissue with numerous cholesterol clefts and associated multicellular giant cells and foamy macrophages, along with the presence of non-keratinized stratified squamous epithelial lining of predominantly two- to four-cell layer thickness, which histologically is indicative of a dentigerous cyst having an inflammatory change [7-9].

A report of a similar case by Abdelkarim et al. [3] revealed a similar odontogenic etiology, creating a void in the understanding of the true etiology of CGs, which suggests that CGs, although associated with the maxillary sinus, can have an odontogenic origin and can occur in either jaw with no distinct radiographic appearance [10-13].

That being said, the treatment protocol for CGs is similar to that of any odontogenic cyst of the oral cavity, i.e., surgical enucleation of the lesion with a minimal chance of recurrence [14].

A literature review done by Richards et al. in 2022 of mastoid cholesteatoma yielded 16 reported cases of CG of mastoid [15]. A comparison with the mastoid variant was made because of its extremely aggressive nature in terms of its bone resorption ability. Although the oral cavity variant of CIS is not as aggressive when left untreated, CGs of the oral cavity show large volumes of bone loss. The treatment protocol was the same as that employed in this case, with surgical enucleation and curettage being the most common treatment modalities. The postoperative follow-up of all 16 cases showed no recurrence or any postoperative complications in the defined review period [15].

## Conclusions

A CG is identified as a histological reaction to cholesterol crystals rather than representing a specific clinical or pathological entity. Given the absence of distinctive clinical and radiographic features, it is crucial to consider central giant cell granuloma as a potential diagnosis for odontogenic cysts and tumors. A precise determination of CGs requires a histological examination.

The precise nature and pathophysiology of documented intraoral CG cases remain uncertain due to their rarity. Ambiguity also exists regarding the terminology and differentiation of CGs observed in the oral cavity and ear. It is imperative to reveal additional instances of oral cavity CGs to elucidate their unique characteristics and provide more comprehensive details.
